# Alcohol and Cannabis Intake in Nursing Students

**DOI:** 10.3390/medicina55100628

**Published:** 2019-09-24

**Authors:** Carlos Tejedor-Cabrera, Omar Cauli

**Affiliations:** 1 Department of Nursing, University of Valencia, 46010 Valencia, Spain; carteca77@gmail.com; 2 Frailty and Cognitive Impairment Group (FROG), University of Valencia, 46010 Valencia, Spain

**Keywords:** alcohol abuse, marijuana, social consequences, CRAFFT scale, audit scale

## Abstract

*Background and objectives:* Drug misuse among young people has become a major worldwide health concern. The present study analyzes substance misuse and its social and personal consequences in young university students. *Materials and Methods:* Screening of alcohol misuse was based on the Alcohol Use Disorder Identification Test (AUDIT), while screening of substance-related risks and problems was performed with the Car, Relax, Alone, Forget, Friends, Trouble (CRAFFT) score. *Results*: The population was composed of nursing students at the University of Valencia (Valencia, Spain) (n = 185). More than 50% of the surveyed students reported alcohol intake based on the CRAFFT scale; 31.4% were classified as having “risky alcohol use”, and 19.5% met the criterion for hazardous drinking based on the AUDIT score. In turn, 34.1% of the sample reported marijuana/hashish intake based on the CRAFFT scale. A gender effect was only observed for marijuana/hashish use, which was significantly (*p* < 0.001) higher in male students. No other gender differences were observed. In the logistic regression analysis, only age was identified as a protective factor for obtaining a reduced risk score with both the AUDIT and the CRAFFT. Among the social and personal consequences of drug misuse, the inability to “stop drinking once you have started” or the inability to “remember what happened while consuming” was significantly associated with an increased frequency of alcohol consumption (OR 20.93, *p* < 0.0001 and OR 13.68, *p* < 0.05, respectively). *Conclusions:* Our findings are consistent with emerging social concerns about drug misuse in the university population, including nursing students as future healthcare professionals.

## 1. Introduction

Drug misuse among young people has become a major worldwide health concern. Experimentation with alcohol and illicit substances begins during adolescence [1]. Over recent decades, health agencies and university authorities have expressed concerns about increasing consumption of alcohol, and also of other drugs of misuse such as cannabis and amphetamines, among university students [2,3,4,5,6,7]. Moreover, alcohol often plays a relevant role in young people’s lives when they enter university [8,9]. The minimum age to consume and to buy alcohol drinks in Spain is 18 years old, and the use of cannabis derivatives for recreational or medical uses is not legalized. Multiple factors contribute to young university student risk-related drug misuse [9,10], but in particular, such students are at a risk of substance misuse because of changes in lifestyle, lessened parental support, and the presence of stressful situations [11]. Higher education studies offered by universities, and specifically in the field of the health sciences, should provide knowledge about the harmful health and social consequences of the use and misuse of alcohol and other drugs of misuse, as these represent major health and social concerns in university life [4]. The consequences of alcohol misuse have been associated with physical health problems and poor academic performance [12,13]. There are comparatively few studies on substance misuse among nursing students in Spain, which is one of the most numerous classes of future healthcare professionals. This is a matter of concern, given that most nursing students will go on to work as nurses and healthcare providers. Personal substance misuse among nurses may impair their future fitness to practice and limit the recognition of problems of substance misuse in their own patients [14,15,16]. In this regard, it is important to detect such problems and adopt strategies to reduce their magnitude during nursing education. Knowledge of the current levels of substance use among nursing students would allow the introduction of preventive actions in this area. Accordingly, the main objectives of the present study were to:

- Examine the intake of alcohol, marijuana/hashish, and other illicit drugs in nursing students.
- Explore the social and personal consequences of substance misuse and the moderating effects of sociodemographic variables.

## 2. Materials and Methods

### 2.1. Design and Participants

A cross-sectional study involving a quantitative approach was carried out. The study involved undergraduate students enrolled in the nursing degree at the University of Valencia (Valencia, Spain) during the academic year 2017–2018. The sole exclusion criterion was refusal to participate after being informed about the objectives of the study. Of the total 245 registered students invited to participate, 185 accepted (75.51%).

The study was anonymous, and permission to survey the students was obtained from the senior medical and law school officials. A member of the research team held a compulsory lecture/seminar to explain the study and distribute the questionnaires. Blank questionnaires and their envelopes were given out to all students present in the teaching sessions. The students were asked to deliver their questionnaire sealed in the envelope to the faculty reception desk, whether completed (students participating in the study) or not (students not participating in the study), thereby further improving anonymity of the study. All questionnaires were kept in a cardboard box. The questionnaire was accompanied by an explanatory opening page about the survey and the eventual distress that the questionnaire could produce. Written consent was sealed in another envelope and was, likewise, returned to the faculty reception desk and kept in the box. Before analyzing the questionnaires, we mixed the content of the box containing the envelopes with the written consents and the envelopes containing the questionnaires (completed and not completed). In this way, the written consents could not be linked to the questionnaires at the time of data collection. Participation in the study was voluntary, and no financial incentive was given. The Ethics Committee of the University of Valencia was consulted to assess the suitability of the research and approved the research design (protocol H1480590883286, dated 21 December 2016). The data collecting instrument was introduced by means of an informative letter about the purposes of the study. Each student decided whether or not to accept participation in the study at the end of the letter, and this was taken to represent written informed consent. Likewise, to preserve data confidentiality and anonymity, the questionnaires were identified by a numerical code. Also, as there was no record of personal data identifying the students, individual responses could not be traced to any specific student.

### 2.2. Sociodemographic Data

We collected basic information related to age, gender, nationality, academic course, and information about working while in school, having children, and place of residency. When a significant result was found for the variable “age” expressed as a discrete variable (quantitative variable), we further analyzed the influence of age by categorizing the variable into a new variable (dichotomic): students <25 years of age and students ≥25 years of age. Such categorization was made because, within the Spanish educational system, the most common ages among university students is under 25 years. Students aged 25 years or older (n = 26 in our sample) are generally students that start their university studies later in life because they work, have previously studied another university degree, or are studying on a part-time basis due to family or work reasons (the latter group included 11 students who were over 30 years old).

### 2.3. Evaluation of Alcohol Consumption

Alcohol consumption was evaluated with the full Spanish version of the Alcohol Consumption Disorders Identification Test (AUDIT) [17]. The AUDIT is a simple screening method developed by the World Health Organization (WHO) to identify a pattern of risky or harmful alcohol consumption, and it has demonstrated reasonable psychometric properties in university students [18,19] with an internal consistency (Cronbach’s alpha) of 0.75, a reliability index of 0.87 (Spearman’s correlation test), and a concordance index of 0.85 (kappa coefficient) [20]. AUDIT is a 10-item scale that evaluates three conceptual domains: hazardous alcohol use (items 1–3), dependence symptoms (items 4–6), and harmful alcohol use (items 7–10). Each of these items is scored from 0 to 4, except for items 9 and 10, which score only 0, 2, or 4. The total scores range from 0 to 40. The greater the number of points, the greater the alcohol dependence. A total AUDIT score of 8 or more was used as the cut-off point for identifying subjects with hazardous and harmful alcohol use, as well as possible alcohol dependence [20,21]. A score of 8 or more is referred to as a positive screen, and is suggestive of an underlying alcohol use disorder. A score of 8–15 is suggestive of hazardous drinking, 16–19 corresponds to harmful drinking, and 20 or more is indicative of dependent drinking [21]. The AUDIT has a sensitivity and specificity of 92% and 94%, respectively [17].

### 2.4. Evaluation of Substance Misuse

The consumption of psychoactive substances other than alcohol was evaluated by the validated Spanish version of the Car, Relax, Alone, Forget, Friends, Trouble (CRAFFT) scale developed by Knight et al. [22,23]. The Spanish version of CRAFFT has good psychometric properties: internal consistency is 0.74 (Cronbach’s alpha), the reliability index is 0.85 (Spearman’ correlation test), and the concordance index is 0.70 (kappa coefficient) [24]. In terms of the sensitivity and specificity of the Spanish version, values of 74.4% and 96.4%, respectively, have been reported [23]. This scale is a brief application instrument that identifies young people at risk of substance misuse. Its name is a mnemonic of the first letters of key words of the 6 questions in part B of the scale (Car, Relax, Alone, Forget, Friends, and Trouble). The CRAFFT questionnaire consists of two parts: Part A comprises three questions about the use of alcohol, marijuana, and other drugs in the last 12 months, while part B comprises 6 questions about problems related to the consumption of such substances. The response format is dichotomous (yes or no). If the answers to the three questions in part A are “no”, only the first question in part B of the questionnaire is asked. Contrarily, if “yes” is answered to any of the three items in part A, part B of the scale is applied. In the case of a negative response (no), a score of zero is assigned, while an affirmative response (yes) is assigned a score of one. To evaluate the instrument, the scores of the 6 items of part B are added together. Scores equal to or greater than two suggest the presence of abusive consumption, which indicates the need to carry out an additional evaluation [24].

### 2.5. Statistical Analysis

The data of each questionnaire were entered in an MS Excel spreadsheet and subsequently exported and analyzed with the SPSS version 25 statistical package (SPSS Inc., Chicago, IL, USA). We first described the characteristics of the participants, with calculations of the distribution of frequencies, proportions, and measures of central tendency (mean) and dispersion (range and standard deviation (SD)), according to the nature of the variables. In the bivariate analysis, the percentage differences between categorical variables were evaluated with the Pearson’s chi-squared test. The normal distribution of each variable was assessed with the Kolmogorov–Smirnov test. Given that none of the variables exhibited a normal distribution, we used nonparametric statistical tools. The differences between groups were analyzed by the Mann–Whitney U-test and the Kruskal–Wallis test, and bivariate correlations between variables were explored using the Spearman correlation test. Lastly, a logistic regression analysis was carried out to predict the risk of consumption of alcohol and other drugs. Statistical significance was considered for *p* < 0.05.

## 3. Results

### 3.1. Study Sample

The study sample (n = 185) was composed of 25 males (13.5%) and 159 females (86%), with missing data in one case (0.5%). The mean age was 21.97 years (SD 5.89) (range 18–55), and the mean body mass index (BMI) was 22.14 kg/m^2^ (SD 3.74) (range 16.67–40.70). The majority of the students were Spanish (n = 172; 93.0%); 89 (48.4%) were in their first or second year of their nursing degree, and 95 (51.6%) were in their third or fourth year. Forty-two students (22.7%) worked during their university studies. In order to analyze the influence of nursing education, we categorized the students based on the first two (first and second) and last two years (third and fourth) of the nursing degree (nursing is a four-year degree in Spain).

### 3.2. Evaluation of Alcohol Consumption

**Figure 1:**
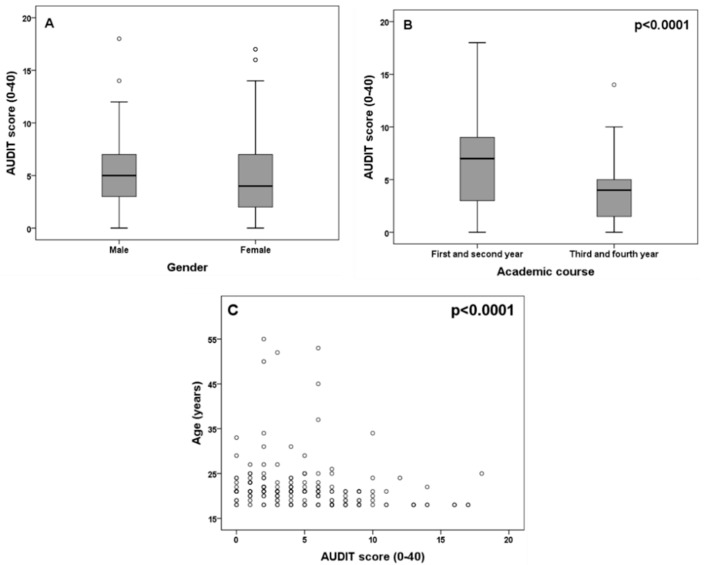
Association between Alcohol Consumption Disorders Identification Test (AUDIT) score and gender (**A**) and academic course (**B**). Plot (**C**) represents the correlation between age and the AUDIT score. Significant *p* values of the Mann–Whitney U-test (**B**) and Spearman correlation test (**C**) are represented in each plot.

The mean AUDIT score was 5.07 (3.88) (range 0–18). On classifying the AUDIT score into the subcategories of hazardous drinking (score 8–15), harmful drinking (16-19) and dependent drinking (≥ 20) [25], we found that 19.5% of the sample showed hazardous drinking and 2.7% harmful drinking. There was no evidence of dependent drinking. There were no gender differences in the overall AUDIT score (*p* = 0.56, Mann–Whitney U-test) (Figure 1A) or in the AUDIT subcategories (*p* = 0.83, chi-squared test).

The mean AUDIT score in the first two years (first and second) of the degree was 6.53 (SD 4.36), while in the last two years (third and fourth) the mean score was 3.71 (SD 2.77). A difference was observed in the AUDIT score between the first and the last two years (*p* < 0.0001, Mann–Whitney U-test) (Figure 1B). There was a significant, inverse correlation between the AUDIT score and age (rho = −0.36, *p* < 0.0001, Spearman correlation test) (Figure 1C). A significant correlation was also observed after excluding students under 30 years of age (rho = −0.37, *p* < 0.0001, Spearman correlation test) or under 25 years of age (rho = −0.40, *p* < 0.0001, Spearman correlation test). No significant correlation was observed between the AUDIT score and BMI (rho = −0.08, *p* = 0.31, Spearman correlation test). Risky alcohol use based on the AUDIT score was identified in 31.4% (n = 58) of the sample, considering a score of 8 or more as the cut-off point for identifying subjects with hazardous and harmful alcohol use. No significant gender differences were observed in relation to risky alcohol use (*p* = 0.91, chi-squared test). A descriptive analysis of the items of the AUDIT scale is represented in the supplementary table.

Fifty percent of the sample had consumed 6 or more drinks on one occasion (item 3). More than 25.9% of the sample scored positively for item 8 (“unable to remember what happened the night before because you had been drinking”). Approximately 20% of the sample scored positively for the item “you were not able to stop drinking once you had started” (item 4) or “you failed to do what was normally expected from you because of drinking” (item 5) at least once a year.

The items that scored negatively in most cases were “have you or someone else been injured as a result of your drinking?” (item 9) (94% of the sample) and “a relative or friend or a doctor or another health worker has been concerned about your drinking” (item 10) (93.5% of the sample). No significant gender differences were observed in any of the items of the AUDIT score (*p* > 0.05, chi-squared test in all cases). There were significant differences in the age of the students in the answer to item 2 (*p* < 0.0001, Kruskal–Wallis test), item 6 (*p* < 0.0001, Kruskal–Wallis test), item 7 (*p* < 0.01, Kruskal–Wallis test), item 8 (*p* < 0.0001, Kruskal–Wallis test), and item 9 (*p* < 0.05, Kruskal–Wallis test).

### 3.3. Evaluation of Substance Misuse and Its Consequences

**Figure 2:**
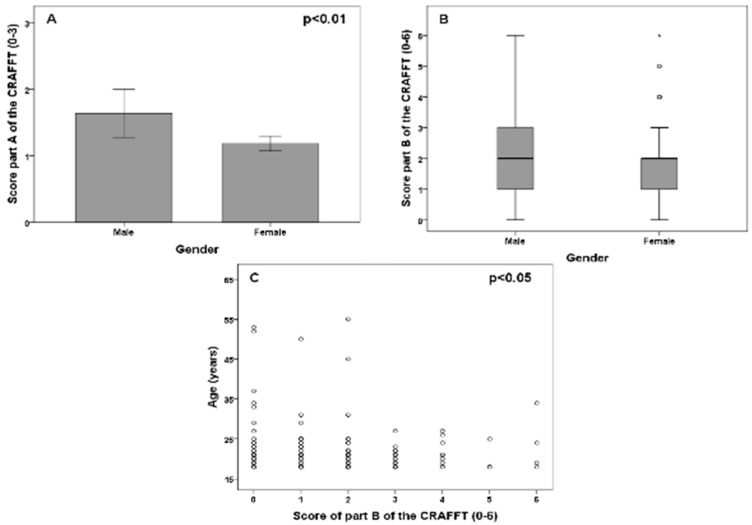
Association between gender and the score in part A of the Car, Relax, Alone, Forget, Friends, Trouble (CRAFFT) scale (**A**) and the score in part B of the CRAFFT scale (**B**). Plot (**C**) represents the correlation between age and the score in part B of the CRAFFT scale. Significant *p* values of the Mann–Whitney U-test (**A**) and Spearman correlation test (**C**) are represented in each plot between AUDIT score and gender (**A**) and academic course (**B**). Plot (**C**) represents the correlation between age and the AUDIT score. Significant *p* values of the Mann–Whitney U-test (**B**) and Spearman correlation test (**C**) are represented in each plot.

The mean value (SD) of the scores in part A of the CRAFFT scale (three questions referred to the consumption of alcohol, marijuana, and other drugs in the last 12 months) was 1.24 (0.75) (range 0–3). Male students had significantly higher scores in part A of the CRAFFT scale compared to female students (*p* < 0.01, Mann–Whitney U-test) (Figure 2A) because of the significantly greater use of marijuana/hashish in male students compared to females (*p* < 0.0001). The mean value of the scores in part A of the CRAFFT scale in the first two years of the degree (first and second) was 1.44 (SD 0.77), while in the last two years (third and fourth) the value was 1.07 (SD 0.67). A difference in part A of the CRAFFT scale was observed between the first and last courses (*p* < 0.001, Mann–Whitney U-test). There was a significant, inverse correlation between part A of the CRAFFT score and age (rho = −0.20, *p* < 0.01, Spearman correlation test). The correlation was also significant in students under 30 years of age (rho = −0.16, *p* < 0.05, Spearman correlation test) and in those under 25 years of age (rho = −0.19, *p* < 0.05, Spearman correlation test). A total of 86.8% of the students consumed alcohol, 34.1% marijuana or hashish, and 5.5% other substances to “get high”. There were no gender differences referred to alcohol use (*p* = 0.79, chi-squared test), though consumption was significantly higher in male students for marijuana/hashish (*p* < 0.01, chi-squared test) and other drugs (*p* < 0.05, chi-squared test).

The mean value (SD) of the scores in part B of the CRAFFT scale (6 questions referred to problems related to the consumption of substances) was 1.72 (1.40) (range 0–6). There were no significant gender differences in the score corresponding to part B of the CRAFFT scale (*p* = 0.25, Mann–Whitney U-test) (Figure 2B). The mean value of the scores in part B of the CRAFFT scale in the first two years of the degree (first and second) was 2.07 (SD 1.57), while in the last two years (third and fourth) the value was 1.41 (SD 1.14). There was a significant difference in part B of the CRAFFT scale between the first and last courses (*p* < 0.01, Mann–Whitney U-test). Likewise, there was a significant, inverse correlation between part B of the CRAFFT scale and age (rho = −0.17, *p* < 0.05, Spearman correlation test) (Figure 2C). The correlation was not significant in students under 30 years of age (rho = −0.13, *p* = 0.08, Spearman correlation test) or in those under 25 years of age (rho = −0.15, *p* = 0.06, Spearman correlation test).

The mean total CRAFFT score (A + B) was 2.96 (1.92) (range 0–9). There were no significant gender differences in the total score of the CRAFFT scale (A + B) (*p* = 0.08, Mann–Whitney U-test). The mean total CRAFFT score in first two years of the degree (first and second) was 3.51 (SD 2.10), while in the last two years (third and fourth) the score was 2.48 (SD 1.59). There was a difference in the total CRAFFT score between the first and last courses (*p* < 0.001, Mann–Whitney U-test). Likewise, a significant, inverse correlation was observed between the total CRAFFT score (A + B) and student age (rho = −0.21, *p* < 0.01, Spearman correlation test). The correlation was also significant in students under 30 years of age (rho = −0.16, *p* < 0.05, Spearman correlation test) and in those under 25 years of age (rho = −0.18, *p* < 0.05, Spearman correlation test).

A descriptive analysis of the items of the CRAFFT scale is represented in Table 1. The most prevalent item with a positive answer was “have you ever been ridden in a car by someone (including yourself) who was “high” or had been using alcohol or drugs?“ (item 4) (75.6% positive answers), followed by the item “do you ever forget things you did while using alcohol or drugs?” (item 8) (52.5% positive answers). A total of 19.4% of the sample consumed alcohol while being alone (item 9). There was a significant and direct correlation between the scores in part A and part B of the CRAFFT scale (rho = 0.54, *p* < 0.0001). A positive response to “how often during the last year have you been unable to stop drinking once you have started?” (item 4) increased the risk referred to frequency of alcohol consumption (item 1) (OR 20.93, 95%CI (4.47–98.04), *p* < 0.0001). The same occurred with the inability to remember what happened while consuming (item 8) (OR 13.68, 95%CI (1.65–113.55), *p* < 0.05, binary logistic regression analysis). None of the variables in part B of the CRAFFT scale increased the risk referred to in items 2 and 3.

### 3.4. Analysis of the Associations between the AUDIT and CRAFFT Scales

**Figure 3:**
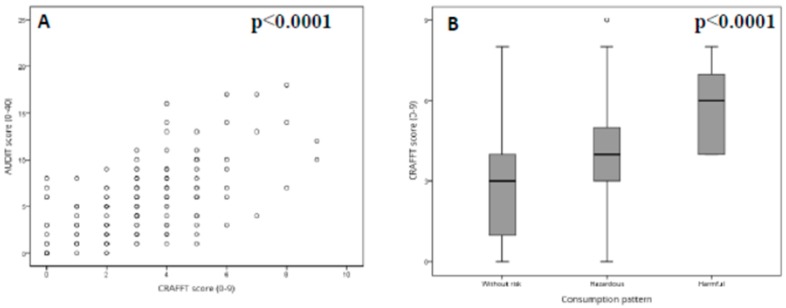
Association between the CRAFFT score and AUDIT score (**A**) and alcohol consumption pattern (**B**). Significant *p* values of the Spearman correlation test (**A**) and Mann–Whitney U-test (**B**) are represented in each plot.

There was a significant, inverse correlation between part A of the CRAFFT score and student age (rho = −0.20, *p* < 0.01, Spearman correlation test). The correlation was also significant in students under 30 years of age (rho = −0.16, *p* < 0.05, Spearman correlation test) and in those under 25 years of age (rho = −0.19, *p* < 0.05, Spearman correlation test). A significant, positive correlation was observed between the total scores of the AUDIT and CRAFFT scales (rho = 0.61, *p* < 0.0001, Spearman correlation test) (Figure 3A). The correlation was also significant and positive between the total scores of the AUDIT and part A of the CRAFFT scale (rho = 0.50, *p* < 0.0001, Spearman correlation test) and between the total scores of the AUDIT and part B of the CRAFFT scale (rho = 0.58, *p* < 0.0001, Spearman correlation test). These associations remained significant when taking into account age, gender, and academic course as confounding variables. There was a significant difference in the total CRAFFT score among the students with risky, harmful, and hazardous alcohol consumption (*p* < 0.0001, Kruskal–Wallis test) (Figure 3B).

Differences were found between the pattern of consumption and the CRAFFT scale in relation to item 4 (*p* < 0.01), item 5 (*p* < 0.0001), item 7 (*p* < 0.001), item 8 (*p* < 0.0001), and item 9 (*p* < 0.01, chi-squared test). However, there were no differences in the pattern of consumption and the use of substances to relax (item 6 of the CRAFFT scale) (*p* = 0.05, chi-squared test). Significant differences were also found between alcohol consumption and the consumption of marijuana/hashish (item 2 of the CRAFFT scale) (*p* < 0.01, chi-squared test). There were no differences between the pattern of alcohol consumption and item 1 (*p* = 0.51, chi-squared test) and item 3 (*p* = 0.68, chi-squared test) of the CRAFFT scale. Students in the last two years of the degree (third and fourth course) had a lower probability of obtaining a risk score in the AUDIT test (OR: 0.194, 95%CI (0.079–0.477), *p* < 0.0001, binary logistic regression analysis). Age (OR 0.887, 95%CI (0.778–1.011), *p* = 0.073, binary logistic regression analysis) and the female gender (OR 0.762, 95%CI (0.236–2.454), *p* = 0.648, binary logistic regression analysis) had no significant impact on the probability of obtaining a risk score in the AUDIT test. However, age was identified as a protective factor against obtaining a risk score in the CRAFFT test (OR: 0.944, 95%CI (0.895–0.996), *p* < 0.05, binary logistic regression analysis). The female gender (OR 0.766, 95%CI (0.261–2.251), *p* = 0.628, binary logistic regression analysis) and being in the last two years of the degree (OR 0.537, 95%CI (0.255–1.131), *p* = 0.102, binary logistic regression analysis) had no significant impact on the probability of obtaining a risk score in the CRAFFT test.

## 4. Discussion

Our study evaluates the drug abuse risk related to alcohol and marijuana/hashish in university students who will become future healthcare providers (nurses), which represents an issue with great social and health implications [26]. Most of the surveyed students reported regular alcohol intake, with one-third being classified as having “risky alcohol use”, while one-fifth met the criterion for hazardous drinking based on the AUDIT score. The reasons underlying the widespread consumption of alcohol and cannabis derivatives in university students have been found to be multifaceted [27] and are attributable to important changes in lifestyle (e.g., leaving the family home to live alone or sharing an apartment with other people, moving to other cities, working and seeking independence in making life decisions, wishing to earn their own money, etc.) [9,10]. In addition, such students endure stress due to the academic workload, pressure to succeed, and competition among peers [11].

A qualitative study using ethnographic open-ended interviews performed among Mexican university students suggested different factors associated with changes in role and status, friendship, and increased autonomy as reasons for their alcohol use after entering university [28]. According to the risk categories proposed by the WHO, more than 30% of the students were classified as having “risky alcohol use” (AUDIT score ≥8), and approximately 20% could be considered hazardous drinkers (AUDIT score ≥16). According to the guidelines for AUDIT screening in primary healthcare, those students with scores between 8–15 are the most appropriate targets for simple advice focused on reducing risk consumption, while in the case of individuals with scores between 16–19, brief therapy and continuous monitoring is advised in addition to simple advice [20]. In turn, AUDIT scores equal to or greater than 20 would require referral to a specialist for a broader diagnostic evaluation of alcohol dependence, though we found no such cases in our study sample. There is some evidence that hazardous drinking in medical school is predictive of later hazardous drinking, thus underscoring the importance of interventions to minimize alcohol intake [29]. Alcohol consumption has traditionally been related to the male gender [26], but no gender differences were found in our sample related to alcohol exposure. This can be attributed to the increase in consumption among women over the last few decades [26]. A study conducted in the United States has reported similar results and suggests that this is due to the social, economic, and role changes that women have achieved in recent years, which, in turn, may increase risk behaviors [29,30]. These results were seen to be reproduced in each of the academic courses, reinforcing the findings of a recent study showing that this trend begins in pre-university stages and continues later on in university academic life [31].

Exposure to cannabis derivatives (marijuana and hashish) was recorded in over 30% of our study sample, representing the second most common drug of abuse consumed by university students [30,32]. In contrast to alcohol exposure, the consumption of marijuana and hashish in our sample showed gender differences, being significantly greater in males compared to females. These results are consistent with those of two surveys among university students that confirm the gender difference in cannabis use [33,34]. A possible explanation for this finding is the greater social rejection of illegal drug use among women [35,36], and the fact that young males are more likely to enter treatment for cannabis misuse [37,38]. The increased prevalence of cannabis use in male students, and the fact that schizophrenia in males develops during adolescence or early adulthood and earlier than in women [39], might suggest a potential role of cannabis during university studies as a potential trigger for schizophrenia, especially in males. Nevertheless, the observed trend is towards similar consumption rates for all substances in both genders [30]. Despite the increasing prevalence of recreational cannabis use among the young population [40] and medical students [41], mainly for its rewarding effects [42,43], the use of cannabinoids among students might also be explained by their anxiolytic, antidepressant, and sleep-promoting effects [44] as a means to mitigate the effects of exposure to different stressors and burnout [11,45,46]. The use of both alcohol and cannabis may affect the current academic performance of students [47], and they may also contribute to misjudgments and misperceptions as future nurses towards patients with substance use disorders [48]. It has been reported that student attitudes towards substance use behaviors influence their future preventive counseling practices [49]. Consequently, the study of lifetime and especially current cannabis use among medical and nursing students is of great importance. This issue is of particular concern for the long-term mental health of the future nursing staff who work under pressure and endure possible employment-related stress that may lead to ongoing use during their professional life, with possible adverse consequences for them and their patients. 

Young people who start their university studies have the highest alcohol consumption rates, even compared to those of the same age who choose other non-university options [50,51,52]. In our sample, the older students (>25 years of age) showed lower rates of alcohol and marijuana/hashish exposure and suffered fewer consequences (remorse or forgetting what happened while consuming, need to drink on fasting to recover, or injuries derived from such consumption), regardless of the academic year. There may be a number of explanations for these observations. In effect, working during university studies could increase responsibility in living independently without the economic coverage of the parents, and it could reduce risky behaviors such as the consumption of these substances [53,54]. On the other hand, the knowledge acquired during academic training might synergize with the effect provided by imminent incorporation to the labor market, thereby also diminishing the pattern of consumption of these substances. Academic training, even at pre-university levels, is positively associated with lower rates of substances use such as alcohol or cannabis [55]. However, unhealthy habits and behaviors do not always improve during the degree, suggesting that nursing studies do not always help to encourage healthy habits [56].

A systematic review concluded that nurses are sufficiently prepared to detect and act on problems related to the consumption of substances such as alcohol [57], and that acquired health knowledge, therefore, also influences risk behaviors. However, the percentage of higher education students who consume alcohol and marijuana/hashish remains much higher than what would be expected from an academic health education such as nursing. A qualitative study carried out in nursing students based on participatory action research methodology concluded that stress, social acceptance, environmental influences, and the availability of alcohol are the most influential factors in the consumption of these substances among nursing students [15]. 

Educational intervention campaigns could be an option for dealing with these problems, employing a set of strategies that have been shown to be effective in changing alcohol-related behaviors to more healthier ones [58]. A study was carried out in the United Kingdom involving a health self-care behavior intervention among new university students, consisting of self-affirmation manipulation, health messages based on the theory of planned behavior, and the implementation of intention tasks. The results showed a significantly lesser use of alcohol among participants in the intervention group than in the control group [59]. Consequently, it is necessary to assess the impact of such health behavior-promoting programs among university students in order to help create a safe and healthy learning environment and promote the development of an integrative health culture. 

Another strategy to minimize drug misuse for stress relief purposes is to learn how to cope with stress. A study by Jensen et al. [60] demonstrated that students who choose coping responses that do not moderate stress where possible, may cause themselves additional distress and prevent the learning of more effective coping responses. Helping students to understand stress and coping, and to develop realistic stress appraisal techniques, may assist students in general to maintain manageable distress levels and functioning [61]. Coping strategies for predicting general health in nursing students have shown promising results for interventions in nurses [62,63]. Students may also need increased curricular knowledge regarding drug misuse from the medical, psychological and social perspectives. In order to respond to all these potential concerns and tailor such interventions, a health promotion office should be available with staff to answer questions and provide needed resources. If such a service already exists on university campuses, increased support should be offered to the staff to allow for the best possible resource availability and the maintenance of anonymity. Creating and supporting such health promotion services may also assist in more efficient collection of data regarding university substance abuse issues and needs.

### Limitations of the Study

The present study was based on a self-reporting method, enhancing the risk of common method bias and potential bias of the responses provided by the participants. In short, we refer to specifically sociological questions addressed by this study, social desirability and acquiescence, and potential gaps involving people participating in self-reporting studies. In this sense, conducting this study in the classroom could involve a certain predisposition to provide the researchers with “positive” or “desirable” answers, even though the lack of wrong or right answers was duly addressed in the introductory phase of the questionnaire.

## 5. Conclusions

In conclusion, we observed an important consumption of alcohol and cannabis-derived products among nursing students. The proportion was higher among the youngest students and was significantly associated with personal and social adverse outcomes such as riding in a car by himself/herself, or someone under the effects of alcohol and other drugs, or with forgetting things while using alcohol or other drugs. Drug misuse-prevention activities should envisage greater protection of university settings, particularly where future health professionals, such as nurses, are involved. Future studies should be undertaken to ascertain the modifiable risk factors that can prevent the consequences of drug misuse at these ages, for example, physical injuries, aggressions, traffic accidents, sexual risk behaviors and emotional problems, or even the development of alcohol dependence.

## Figures and Tables

**Table 1:** Behaviors related to the consumption of alcohol and other substances (items of the CRAFFT scale). The frequency and percentage of each question of the CRAFFT is reported.

	Item	Answer	Frequency	Percentage
PART A	Item 1. Drink any alcohol (more than a few sips)? (Do not count sips of alcohol taken during family or religious events)	Yes	158	86.8
		No	24	13.2
	Item 2. Smoke any marijuana or hashish?	Yes	62	34.1
		No	120	65.9
	Item 3. Use anything else to get high? (“anything else” includes illegal drugs, over the counter and prescription drugs, and things that you sniff or “huff”)	Yes	10	5.5
		No	172	94.5
PART B	Item 4. Have you ever ridden in a car with someone (including yourself) who was “high” or had been using alcohol or drugs?	Yes	121	75.6
		No	39	24.4
	Item 5. Do your family or friends ever tell you that you should cut down on your drinking or drug use?	Yes	17	10.6
		No	143	89.4
	Item 6. Do you ever use alcohol or drugs to relax, feel better about yourself, or fit in?	Yes	48	30.0
		No	112	70.0
	Item 7. Have you ever gotten into trouble while you were using alcohol or drugs?	Yes	17	10.6
		No	143	89.4
	Item 8. Do you ever forget things you did while using alcohol or drugs?	Yes	84	52.5
		No	76	47.5
	Item 9. Do you ever use alcohol or drugs while you are by yourself, or alone?	Yes	31	19.4
		No	129	80.6

## Supplementary Materials

The following are available online at https://www.mdpi.com/1010-660X/55/10/628/s1, Table S1: Descriptive analysis of the items of AUDIT; Table S2: Descriptive analysis of the items of CRAFFT.
