# Supplementary material for: Alcohol and Cannabis Intake in Nursing Students
Source: Medicina (Kaunas). 2019 Sep 24;55(10):628. doi: 10.3390/medicina55100628 (PMC6843613; doi:10.3390/medicina55100628)
Supplement: Supplementary file 1 [file medicina-55-00628-s001.pdf]

medicina

MDPI

Table S1. Descriptive analysis of the items of AUDIT.

Item	Answer	Frecuency (n)	Percentage
Item 1. How often do you have a drink containing alcohol?	Never	18	9.8
	Monthly or less	41	22.3
	2 to 4 times a month	97	52.7
	2 to 3 times a week	27	14.7
	4 or more times a week	1	0.5
Item 2. How many drinks containing alcohol do you have on a typical day when you are drinking?	1 or 2	101	54.9
	3 or 4	50	27.2
	5 or 6	25	13.6
	7, 8 or 9	7	3.8
	10 or more	1	0.5
Item 3. How often do you have six or more drinks on one occasion?	Never	92	50.0
	Less than monthly	59	32.1
	Monthly	32	17.4
	Weekly	1	0.5
	Daily or almost daily	0	0.0
Item 4. How often during the last year have you found that you were no table to stop drinking once you had started?	Never	145	78.8
	Less than monthly	24	13.0
	Monthly	9	4.9
	Weekly	1	0.5
	Daily or almost daily	5	2.7
Item 5. How often during the last year have you failed to do what was normally expected from you because of drinking?	Never	149	81.0
	Less than monthly	31	16.8
	Monthly	4	2.2
	Weekly	0	0.0
	Daily or almost daily	0	0.0
Item 6. How often during the last year have you needed a first drink in the morning to get yourself going after a heavy drinking session?	Never	133	72.3
	Less than monthly	39	21.2
	Monthly	10	5.4
	Weekly	1	0.5
	Daily or almost daily	1	0.5
Item 7. How often during the last year have you had a feeling of guilt or remorse after drinking?	Never	124	67.4
	Less than monthly	43	23.4
	Monthly	13	7.1
	Weekly	4	2.2
	Daily or almost daily	0	0.0
Item 8. How often during the last year have you been unable to remember what happened the night before because you had been drinking?	Never	118	64.1
	Less than monthly	56	30.4
	Monthly	8	4.3
	Weekly	2	1.1
	Daily or almost daily	0	0.0
Item 9. Have you or someone else been injured as a result of your drinking?	No	173	94.0
	Yes, but not in the last year	6	3.3
	Yes, during the last year	5	2.7
Item 10. Has a relative or friend or a doctor or another health worker been concerned about your drinking or suggested you cut down?	No	172	93.5
	Yes, but not in the last year	7	3.8
	Yes, during the last year	5	2.7

Table S2. Descriptive analysis of the items of CRAFFT.

	Item	Answer	Frecuency	Percentage
PART A	Item 1. Drink any alcohol (more than a few sips)? (Do not count sips of alcohol taken during family or religious events)	Yes	158	86.8
		No	24	13.2
	Item 2. Smoke any marijuana or hashish?	Yes	62	34.1
		No	120	65.9
	Item 3. Use anything else to get high? (“anything else” includes illegal drugs, over the counter and prescription drugs, and things that you sniff or “huff”)	Yes	10	5.5
		No	172	94.5
PART B	Item 4. Have you ever ridden in a car by someone (including yourself) who was “high” or had been using alcohol or drugs?	Yes	121	75.6
		No	39	24.4
	Item 5. Do your family or friends ever tell you that you should cut down on your drinking or drug use?	Yes	17	10.6
		No	143	89.4
	Item 6. Do you ever use alcohol or drugs to relax, feel better about yourself, or fit in?	Yes	48	30.0
		No	112	70.0
	Item 7. Have you ever gotten into trouble while you were using alcohol or drugs?	Yes	17	10.6
		No	143	89.4
	Item 8. Do you ever forget things you did while using alcohol or drugs?	Yes	84	52.5
		No	76	47.5
	Item 9. Do you ever use alcohol or drugs while you are by yourself, or alone?	Yes	31	19.4
		No	129	80.6
